# *N*′-(5-Bromo-2-hydroxy­benzyl­idene)-3,4,5-trihydroxy­benzohydrazide dihydrate

**DOI:** 10.1107/S1600536808022708

**Published:** 2008-07-23

**Authors:** Abeer A. Alhadi, Hapipah M. Ali, Subramaniam Puvaneswary, Ward T. Robinson, Seik Weng Ng

**Affiliations:** a Department of Chemistry, University of Malaya, 50603 Kuala Lumpur, Malaysia

## Abstract

The title compound, C_14_H_11_BrN_2_O_5_·2H_2_O, crystallizes as hydrogen-bonded sheets. The 2-hydr­oxy group on the benzyl­idene group forms an intra­molecular hydrogen bond to the N atom of the C=N double bond. The amino N atom is a hydrogen-bond donor to a water mol­ecule. The hydr­oxy group on the benzohydrazide group is a hydrogen-bond donor to one acceptor site, whereas each water mol­ecule is a hydrogen-bond donor to two acceptor sites.

## Related literature

For the structure of a similar Schiff-base ligand, 5-bromo­salicylaldehyde benzoyl­hydrazone, see: Liu *et al.* (2006 ▶).
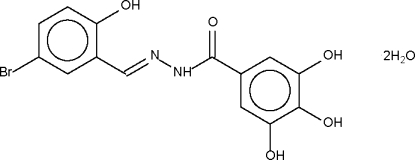


## Experimental

### 

#### Crystal data

C_14_H_11_BrN_2_O_5_·2H_2_O
*M* *_r_* = 403.19
Monoclinic, 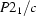

*a* = 30.8424 (8) Å
*b* = 3.7999 (1) Å
*c* = 12.8484 (4) Å
β = 90.280 (2)°
*V* = 1505.79 (7) Å^3^
*Z* = 4
Mo *K*α radiation
μ = 2.77 mm^−1^
*T* = 100 (2) K
0.30 × 0.03 × 0.03 mm

#### Data collection

Bruker SMART APEX diffractometer
Absorption correction: multi-scan (*SADABS*; Sheldrick, 1996 ▶) *T*_min_ = 0.658, *T*_max_ = 0.921
9964 measured reflections
3424 independent reflections
2914 reflections with *I* > 2σ(*I*)
*R*_int_ = 0.039

#### Refinement

*R*[*F* ^2^ > 2σ(*F* ^2^)] = 0.064
*wR*(*F* ^2^) = 0.155
*S* = 1.22
3424 reflections
241 parameters
10 restraints
H atoms treated by a mixture of independent and constrained refinement
Δρ_max_ = 1.08 e Å^−3^
Δρ_min_ = −1.82 e Å^−3^

### 

Data collection: *APEX2* (Bruker, 2007 ▶); cell refinement: *SAINT* (Bruker, 2007 ▶); data reduction: *SAINT*; program(s) used to solve structure: *SHELXS97* (Sheldrick, 2008 ▶); program(s) used to refine structure: *SHELXL97* (Sheldrick, 2008 ▶); molecular graphics: *X-SEED* (Barbour, 2001 ▶); software used to prepare material for publication: *publCIF* (Westrip, 2008 ▶).

## Supplementary Material

Crystal structure: contains datablocks global, I. DOI: 10.1107/S1600536808022708/bt2751sup1.cif

Structure factors: contains datablocks I. DOI: 10.1107/S1600536808022708/bt2751Isup2.hkl

Additional supplementary materials:  crystallographic information; 3D view; checkCIF report

## Figures and Tables

**Table 1:** Hydrogen-bond geometry (Å, °)

*D*—H⋯*A*	*D*—H	H⋯*A*	*D*⋯*A*	*D*—H⋯*A*
O1—H1*o*⋯N1	0.84 (1)	1.91 (5)	2.616 (6)	141 (7)
O3—H3*o*⋯O2*w*	0.84 (1)	1.96 (4)	2.736 (6)	153 (7)
O4—H4*o*⋯O2*w*^i^	0.84 (1)	1.81 (3)	2.623 (8)	163 (9)
O5—H5*o*⋯O2^ii^	0.84 (1)	1.93 (2)	2.764 (5)	171 (7)
O1*w*—H1*w*1⋯O2^iii^	0.84 (1)	1.98 (2)	2.812 (5)	170 (6)
O1*w*—H1*w*2⋯O1^ii^	0.84 (1)	2.09 (2)	2.914 (6)	167 (6)
O2*w*—H2*w*1⋯O3^iv^	0.84 (1)	2.13 (5)	2.845 (9)	142 (8)
O2*w*—H2*w*2⋯O4^v^	0.84 (1)	2.12 (4)	2.900 (8)	154 (8)

Symmetry codes: (i) 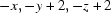; (ii) 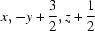; (iii) 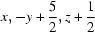; (iv) 
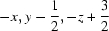
; (v) 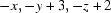.

**Fig. 1.**
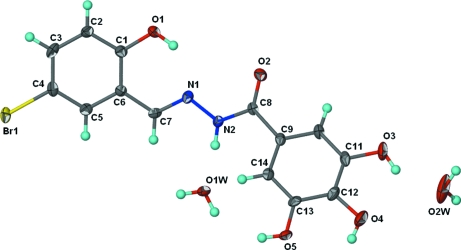
View (Barbour, 2001) of N-(5-bromo-2-hydroxybenzylidene)-3,4,5-trihydroxybenzohydrazide with displacement ellipsoids at the 70% probability level. Hydrogen atoms are drawn as spheres of arbitrary radius.

## supplementary crystallographic information

### Comment

This study extends the structural study on the Schiff base, 5-bromosalicylaldehyde benzoylhydrazone (Liu *et al.*, 2006) as the title compound (Scheme I, Fig. 1) has several hydroxy groups on one of the aromatic rings. The compound crystallizes with two lattice water molecules. Hydrogen bonding interactions (Table 1) give rise to a layer motif.

### Experimental

3,4,5-Trihydroxybenzoylhydrazide (0.65 g, 3.5 mmol) and 5-bromo-2-hydroxybenzaldehyde (0.70 g, 3.5 mmol) were heated for 12 h in ethanol. The solvent was removed and the product recrystallized from ethanol.

### Refinement

Carbon and nitrogen-bound H-atoms were placed in calculated positions (C—H 0.95 Å; N–H 0.88 Å) and were included in the refinement in the riding model approximation, with *U*_iso_(H) 1.2 *U*_eq_(C). The hydroxy and water H-atoms were located in a difference Fourier map, and were refined with distance restraints of O–H 0.84±0.01 Å and H···H 1.37±0.01 Å.

The final difference Fourier map had a   peak of 1.37eÅ^-3^ at 0.69Å from Br1 and a hole of -1.81eÅ^-3^ at 1.33Å from C2.

### Crystal data

**Table:**

C_14_H_11_BrN_2_O_5_·2H_2_O	*F*_000_ = 816
*M**_r_* = 403.19	*D*_x_ = 1.779 Mg m^−^^3^
Monoclinic, *P*2_1_/*c*	Mo *K*α radiation λ = 0.71073 Å
Hall symbol: -P 2ybc	Cell parameters from 2758 reflections
*a* = 30.8424 (8) Å	θ = 3.2–27.4º
*b* = 3.7999 (1) Å	µ = 2.77 mm^−^^1^
*c* = 12.8484 (4) Å	*T* = 100 (2) K
β = 90.280 (2)º	Needle, colorless
*V* = 1505.79 (7) Å^3^	0.30 × 0.03 × 0.03 mm
*Z* = 4	

### Data collection

**Table:**

Bruker SMART APEX diffractometer	3424 independent reflections
Radiation source: fine-focus sealed tube	2914 reflections with *I* > 2σ(*I*)
Monochromator: graphite	*R*_int_ = 0.039
*T* = 100(2) K	θ_max_ = 27.5º
ω scans	θ_min_ = 1.3º
Absorption correction: Multi-scan(SADABS; Sheldrick, 1996)	*h* = −38→40
*T*_min_ = 0.658, *T*_max_ = 0.921	*k* = −4→4
9964 measured reflections	*l* = −15→16

### Refinement

**Table:**

Refinement on *F*^2^	Secondary atom site location: difference Fourier map
Least-squares matrix: full	Hydrogen site location: inferred from neighbouring sites
*R*[*F*^2^ > 2σ(*F*^2^)] = 0.064	H atoms treated by a mixture of independent and constrained refinement
*wR*(*F*^2^) = 0.155	*w* = 1/[σ^2^(*F*_o_^2^) + (0.0499*P*)^2^ + 10.2476*P*] where *P* = (*F*_o_^2^ + 2*F*_c_^2^)/3
*S* = 1.22	(Δ/σ)_max_ = 0.001
3424 reflections	Δρ_max_ = 1.08 e Å^−^^3^
241 parameters	Δρ_min_ = −1.82 e Å^−^^3^
10 restraints	Extinction correction: none
Primary atom site location: structure-invariant direct methods	

### Special details

**Table:**

Geometry. All e.s.d.'s (except the e.s.d. in the dihedral angle between two l.s. planes) are estimated using the full covariance matrix. The cell e.s.d.'s are taken into account individually in the estimation of e.s.d.'s in distances, angles and torsion angles; correlations between e.s.d.'s in cell parameters are only used when they are defined by crystal symmetry. An approximate (isotropic) treatment of cell e.s.d.'s is used for estimating e.s.d.'s involving l.s. planes.

### Fractional atomic coordinates and isotropic or equivalent isotropic displacement parameters (Å^2^)

**Table:**

	*x*	*y*	*z*	*U*_iso_*/*U*_eq_	
Br1	0.467910 (16)	0.31017 (16)	0.89799 (4)	0.01787 (17)	
O1	0.32358 (12)	0.6446 (12)	0.5944 (3)	0.0201 (9)	
H1O	0.3020 (15)	0.745 (18)	0.621 (5)	0.030*	
O2	0.20078 (11)	0.9755 (11)	0.6545 (3)	0.0182 (8)	
O3	0.05472 (13)	1.3768 (13)	0.7962 (4)	0.0295 (11)	
H3O	0.0335 (16)	1.36 (2)	0.836 (5)	0.044*	
O4	0.05208 (14)	1.1036 (15)	0.9894 (4)	0.0353 (12)	
H4O	0.048 (3)	0.933 (15)	1.030 (6)	0.053*	
O5	0.12324 (12)	0.7692 (12)	1.0737 (3)	0.0182 (9)	
H5O	0.1471 (12)	0.682 (18)	1.092 (5)	0.027*	
O1W	0.25912 (12)	1.3152 (13)	0.9988 (3)	0.0209 (9)	
H1W1	0.2444 (17)	1.382 (18)	1.050 (3)	0.031*	
H1W2	0.2806 (14)	1.199 (17)	1.020 (4)	0.031*	
O2W	−0.02887 (15)	1.3435 (17)	0.8657 (5)	0.0506 (16)	
H2W1	−0.042 (3)	1.30 (2)	0.810 (4)	0.076*	
H2W2	−0.038 (3)	1.534 (14)	0.890 (7)	0.076*	
N1	0.27850 (13)	0.8683 (12)	0.7531 (3)	0.0139 (9)	
N2	0.24174 (13)	0.9859 (13)	0.8027 (3)	0.0138 (9)	
H2N	0.2431	1.0511	0.8684	0.017*	
C1	0.35563 (16)	0.5778 (15)	0.6648 (4)	0.0144 (10)	
C2	0.39381 (16)	0.4251 (16)	0.6295 (4)	0.0173 (11)	
H2	0.3970	0.3730	0.5576	0.021*	
C3	0.42746 (17)	0.3479 (16)	0.6985 (4)	0.0193 (12)	
H3	0.4535	0.2431	0.6743	0.023*	
C4	0.42245 (16)	0.4256 (16)	0.8025 (4)	0.0160 (11)	
C5	0.38470 (16)	0.5781 (15)	0.8398 (4)	0.0143 (10)	
H5	0.3820	0.6294	0.9118	0.017*	
C6	0.35065 (15)	0.6562 (15)	0.7712 (4)	0.0126 (10)	
C7	0.31105 (16)	0.7988 (15)	0.8138 (4)	0.0130 (10)	
H7	0.3089	0.8415	0.8865	0.016*	
C8	0.20343 (16)	1.0010 (13)	0.7503 (4)	0.0108 (10)	
C9	0.16507 (16)	1.0460 (15)	0.8180 (4)	0.0130 (10)	
C10	0.12793 (16)	1.2046 (16)	0.7771 (4)	0.0163 (11)	
H10	0.1281	1.2984	0.7086	0.020*	
C11	0.09101 (17)	1.2242 (15)	0.8368 (4)	0.0176 (12)	
C12	0.09010 (17)	1.0775 (17)	0.9364 (4)	0.0204 (12)	
C13	0.12713 (17)	0.9146 (15)	0.9777 (4)	0.0152 (11)	
C14	0.16479 (17)	0.9040 (15)	0.9188 (4)	0.0149 (11)	
H14	0.1903	0.8006	0.9468	0.018*	

### Atomic displacement parameters (Å^2^)

**Table:**

	*U*^11^	*U*^22^	*U*^33^	*U*^12^	*U*^13^	*U*^23^
Br1	0.0094 (2)	0.0195 (3)	0.0247 (3)	−0.0001 (2)	−0.00302 (17)	−0.0020 (3)
O1	0.0160 (18)	0.032 (3)	0.0125 (17)	0.0031 (18)	−0.0028 (14)	−0.0016 (17)
O2	0.0127 (17)	0.027 (2)	0.0148 (18)	−0.0039 (16)	−0.0015 (14)	0.0021 (17)
O3	0.0142 (19)	0.041 (3)	0.034 (2)	0.0120 (19)	−0.0051 (17)	−0.005 (2)
O4	0.016 (2)	0.060 (4)	0.030 (2)	0.009 (2)	0.0035 (18)	−0.013 (2)
O5	0.0129 (17)	0.031 (2)	0.0110 (17)	−0.0007 (17)	0.0027 (14)	−0.0045 (16)
O1W	0.0162 (18)	0.035 (2)	0.0113 (17)	0.0027 (18)	0.0015 (14)	−0.0044 (18)
O2W	0.016 (2)	0.049 (4)	0.087 (5)	0.010 (2)	0.010 (2)	0.013 (3)
N1	0.0107 (19)	0.014 (3)	0.017 (2)	−0.0016 (17)	0.0010 (16)	−0.0008 (18)
N2	0.0096 (19)	0.019 (3)	0.013 (2)	−0.0004 (18)	−0.0007 (15)	−0.0028 (18)
C1	0.014 (2)	0.016 (3)	0.014 (2)	−0.002 (2)	−0.0013 (19)	−0.002 (2)
C2	0.013 (2)	0.025 (3)	0.014 (2)	−0.001 (2)	0.0024 (19)	0.000 (2)
C3	0.013 (2)	0.020 (3)	0.025 (3)	0.001 (2)	0.006 (2)	−0.003 (2)
C4	0.012 (2)	0.014 (3)	0.021 (3)	−0.001 (2)	−0.003 (2)	0.003 (2)
C5	0.015 (2)	0.013 (3)	0.015 (2)	−0.001 (2)	0.0005 (19)	−0.002 (2)
C6	0.010 (2)	0.015 (3)	0.013 (2)	−0.003 (2)	0.0008 (18)	0.001 (2)
C7	0.013 (2)	0.015 (3)	0.012 (2)	−0.006 (2)	0.0022 (18)	−0.003 (2)
C8	0.013 (2)	0.004 (3)	0.015 (2)	−0.0007 (18)	−0.0019 (18)	0.0009 (19)
C9	0.011 (2)	0.013 (3)	0.015 (2)	0.000 (2)	−0.0027 (19)	−0.003 (2)
C10	0.013 (2)	0.016 (3)	0.020 (3)	0.001 (2)	−0.0034 (19)	0.000 (2)
C11	0.017 (2)	0.013 (3)	0.022 (3)	0.008 (2)	−0.007 (2)	−0.007 (2)
C12	0.012 (2)	0.027 (3)	0.022 (3)	0.002 (2)	0.001 (2)	−0.012 (2)
C13	0.016 (2)	0.017 (3)	0.013 (2)	0.001 (2)	0.0025 (19)	−0.008 (2)
C14	0.013 (2)	0.016 (3)	0.015 (2)	0.001 (2)	−0.0022 (19)	−0.006 (2)

### Geometric parameters (Å, °)

**Table:**

Br1—C4	1.909 (5)	C1—C6	1.408 (7)
O1—C1	1.361 (6)	C2—C3	1.393 (8)
O1—H1O	0.840 (10)	C2—H2	0.9500
O2—C8	1.236 (6)	C3—C4	1.378 (8)
O3—C11	1.362 (6)	C3—H3	0.9500
O3—H3O	0.838 (10)	C4—C5	1.388 (7)
O4—C12	1.362 (7)	C5—C6	1.400 (7)
O4—H4O	0.840 (10)	C5—H5	0.9500
O5—C13	1.357 (7)	C6—C7	1.446 (7)
O5—H5O	0.841 (10)	C7—H7	0.9500
O1W—H1W1	0.839 (10)	C8—C9	1.482 (7)
O1W—H1W2	0.838 (10)	C9—C10	1.395 (7)
O2W—H2W1	0.838 (10)	C9—C14	1.403 (7)
O2W—H2W2	0.839 (10)	C10—C11	1.378 (7)
N1—C7	1.296 (7)	C10—H10	0.9500
N1—N2	1.378 (6)	C11—C12	1.396 (8)
N2—C8	1.359 (6)	C12—C13	1.401 (8)
N2—H2N	0.8800	C13—C14	1.390 (7)
C1—C2	1.391 (7)	C14—H14	0.9500
			
C1—O1—H1O	113 (5)	C5—C6—C7	118.3 (4)
C11—O3—H3O	111 (6)	C1—C6—C7	122.9 (5)
C12—O4—H4O	112 (6)	N1—C7—C6	120.1 (4)
C13—O5—H5O	110 (5)	N1—C7—H7	120.0
H1W1—O1W—H1W2	109.6 (18)	C6—C7—H7	120.0
H2W1—O2W—H2W2	109.5 (18)	O2—C8—N2	122.9 (4)
C7—N1—N2	115.1 (4)	O2—C8—C9	123.0 (4)
C8—N2—N1	120.0 (4)	N2—C8—C9	114.1 (4)
C8—N2—H2N	120.0	C10—C9—C14	120.3 (5)
N1—N2—H2N	120.0	C10—C9—C8	119.0 (5)
O1—C1—C2	118.3 (5)	C14—C9—C8	120.4 (5)
O1—C1—C6	121.6 (5)	C11—C10—C9	119.5 (5)
C2—C1—C6	120.1 (5)	C11—C10—H10	120.2
C1—C2—C3	120.6 (5)	C9—C10—H10	120.2
C1—C2—H2	119.7	O3—C11—C10	119.3 (5)
C3—C2—H2	119.7	O3—C11—C12	120.1 (5)
C4—C3—C2	119.1 (5)	C10—C11—C12	120.6 (5)
C4—C3—H3	120.5	O4—C12—C11	116.7 (5)
C2—C3—H3	120.5	O4—C12—C13	123.0 (5)
C3—C4—C5	121.5 (5)	C11—C12—C13	120.3 (5)
C3—C4—Br1	119.2 (4)	O5—C13—C14	124.1 (5)
C5—C4—Br1	119.3 (4)	O5—C13—C12	116.7 (5)
C4—C5—C6	120.0 (5)	C14—C13—C12	119.2 (5)
C4—C5—H5	120.0	C13—C14—C9	120.0 (5)
C6—C5—H5	120.0	C13—C14—H14	120.0
C5—C6—C1	118.7 (5)	C9—C14—H14	120.0
			
C7—N1—N2—C8	−167.2 (5)	N2—C8—C9—C10	154.0 (5)
O1—C1—C2—C3	−179.3 (5)	O2—C8—C9—C14	147.8 (5)
C6—C1—C2—C3	0.1 (9)	N2—C8—C9—C14	−31.4 (7)
C1—C2—C3—C4	−0.1 (9)	C14—C9—C10—C11	0.6 (9)
C2—C3—C4—C5	0.1 (9)	C8—C9—C10—C11	175.2 (5)
C2—C3—C4—Br1	178.5 (4)	C9—C10—C11—O3	−179.5 (5)
C3—C4—C5—C6	0.0 (9)	C9—C10—C11—C12	−2.0 (9)
Br1—C4—C5—C6	−178.4 (4)	O3—C11—C12—O4	−1.4 (8)
C4—C5—C6—C1	−0.1 (8)	C10—C11—C12—O4	−178.9 (6)
C4—C5—C6—C7	177.0 (5)	O3—C11—C12—C13	178.9 (5)
O1—C1—C6—C5	179.4 (5)	C10—C11—C12—C13	1.4 (9)
C2—C1—C6—C5	0.1 (8)	O4—C12—C13—O5	2.2 (9)
O1—C1—C6—C7	2.5 (9)	C11—C12—C13—O5	−178.1 (5)
C2—C1—C6—C7	−176.9 (5)	O4—C12—C13—C14	−179.1 (6)
N2—N1—C7—C6	176.8 (5)	C11—C12—C13—C14	0.6 (9)
C5—C6—C7—N1	−178.7 (5)	O5—C13—C14—C9	176.7 (5)
C1—C6—C7—N1	−1.8 (8)	C12—C13—C14—C9	−1.9 (8)
N1—N2—C8—O2	−13.6 (8)	C10—C9—C14—C13	1.3 (8)
N1—N2—C8—C9	165.7 (5)	C8—C9—C14—C13	−173.2 (5)
O2—C8—C9—C10	−26.7 (8)		

### Hydrogen-bond geometry (Å, °)

**Table:**

*D*—H···*A*	*D*—H	H···*A*	*D*···*A*	*D*—H···*A*
O1—H1o···N1	0.84 (1)	1.91 (5)	2.616 (6)	141 (7)
O3—H3o···O2w	0.84 (1)	1.96 (4)	2.736 (6)	153 (7)
O4—H4o···O2w^i^	0.84 (1)	1.81 (3)	2.623 (8)	163 (9)
O5—H5o···O2^ii^	0.84 (1)	1.93 (2)	2.764 (5)	171 (7)
O1w—H1w1···O2^iii^	0.84 (1)	1.98 (2)	2.812 (5)	170 (6)
O1w—H1w2···O1^ii^	0.84 (1)	2.09 (2)	2.914 (6)	167 (6)
O2w—H2w1···O3^iv^	0.84 (1)	2.13 (5)	2.845 (9)	142 (8)
O2w—H2w2···O4^v^	0.84 (1)	2.12 (4)	2.900 (8)	154 (8)

Symmetry codes: (i) −*x*, −*y*+2, −*z*+2; (ii) *x*, −*y*+3/2, *z*+1/2; (iii) *x*, −*y*+5/2, *z*+1/2; (iv) −*x*, *y*−1/2, −*z*+3/2; (v) −*x*, −*y*+3, −*z*+2.
